# Embedding similarities between embryos and circulating tumor cells: fundamentals of abortifacients used for cancer metastasis chemoprevention

**DOI:** 10.1186/s13046-021-02104-4

**Published:** 2021-09-23

**Authors:** Jie Wang, Xiaobo Yu, Huayi Peng, Yusheng Lu, Shuhui Li, Qing Shi, Jian Liu, Haiyan Dong, Vladimir Katanaev, Lee Jia

**Affiliations:** 1 College of Materials and Chemical Engineering, Minjiang University, 350108 Fuzhou, China; 2 Inflammation and Immune Mediated Diseases Laboratory of Anhui Province, Anhui Institute of Innovative Drugs, Institute for Liver Diseases of Anhui Medical University, School of Pharmacy, Anhui Medical University, Hefei, China; 3 Cancer Metastasis Alert and Prevention Center, College of Chemistry; Fujian Provincial Key Laboratory of Cancer Metastasis Chemoprevention and Chemotherapy, Fuzhou University, 350108 Fuzhou, P.R. China; 4 Fujian Provincial Key Laboratory of Inspection and Quarantine Technology Research/ Technology Center of Fuzhou Customs, 350108 Fuzhou, China; 5 Fujian Key Laboratory for Translational Research in Cancer and Neurodegenerative Diseases Institute for Translational Medicine, School of Basic Medical Sciences, Fujian Medical University, 350108 Fuzhou, China; 6 Translational Research Center in Oncohaematology, Department of Cell Physiology and Metabolism, Faculty of Medicine, University of Geneva, Geneva, Switzerland

**Keywords:** Circulating tumor cells, Cancer metastatic chemoprevention, Abortifacients, Embryo implantation, Adhesion/invasion

## Abstract

**Background:**

The global epidemiological studies reported lower cancer risk after long-term use of contraceptives. Our systematic studies demonstrated that abortifacients are effective in preventing cancer metastases induced by circulating tumor cells (CTCs). However, the molecular and cellular mechanisms by which abortifacients prevent CTC-based cancer metastases are almost unknown. The present studies were designed to interdisciplinarily explore similarities and differences between embryo implantation and cancer cell adhesion/invasion.

**Methods:**

Biomarker expressions on the seeding embryo JEG-3 and cancer MCF-7 cells, as well as embedding uterine endometrial RL95-2 and vascular endothelial HUVECs cells were examined and compared before and after treatments with 17β-estradiol plus progesterone and abortifacients. Effects of oral metapristone and mifepristone on embryo implantation in normal female mice and adhesion/invasion of circulating tumor cells (CTCs) in BALB/C female mice were examined.

**Results:**

Both embryo JEG-3 and cancer MCF-7 cells expressed high sLex, CD47, CAMs, while both endometrial RL95-2 and endothelial HUVECs exhibited high integrins and ICAM-1. Near physiological concentrations of 17β-estradiol plus progesterone promoted migration and invasion of JEG-3 and MCF-7 cells via upregulating integrins and MMPs. Whereas, mifepristone and metapristone significantly inhibited migration and invasion of JEG-3 and MCF-7 cells, and inhibited JEG-3 and MCF-7 adhesion to matrigel, RL95-2 cells and HUVECs, respectively. The inhibitions were realized by downregulating sLex, MMPs in JEG-3 and MCF-7 cells, and downregulating integrins in RL95-2 cells and HUVECs, respectively. Mifepristone and metapristone significantly inhibited both embryo implantation and cancer cell metastasis in mice.

**Conclusions:**

The similarities between the two systems provide fundamentals for abortifacients to intervene CTC adhesion/invasion to the distant metastatic organs. The present studies offer the rationale to repurpose abortifacients for safe and effective cancer metastasis chemoprevention.

## Background

The global cancer theranostics is witnessing today’s success of primary cancer treatments and today’s failure of post-metastasis chemotherapy [1]. We have to admit that the anti-cancer drugs can hardly stop the cancer metastasis cascade that already spreads over the body, and the death due to metastasis cannot be reversed. Therefore, a new strategy for cancer treatment must be developed to win the cancer war.

Before GLOBOCAN estimated 19.3 million new cancer cases and almost 10.0 million cancer deaths worldwide occurred in 2020 for 36 Cancers in 185 countries [2], Lancet Oncology in 2015 published a large epidemiological study [3] that indicated that long-term administration of oral contraceptives prevented the risks of ovarian cancer and endometrial cancer. The study involved 27,000 more women from 36 epidemiological studies and concluded that use of oral contraceptives confers long-term protection against endometrial cancer. In short, the most important finding of the study is that the longer the women had used oral contraceptives, the greater the reduction in ovarian cancer risk. This reduction in cancer risk persisted for more than 30 years after oral contraceptive use had ceased but became somewhat weakened over time: the proportional risk reductions per 5 years of use were 29% for use that had ceased less than 10 years previously, 19% for use that had ceased 10–19 years previously, and 15% for use that had ceased 20–29 years previously.

Inspired by the large epidemiological results, we hypothesized that if an abortifacient could interfere with implantation of the fertilized egg (blastocyst) into endometrium, and lead to infertility, the abortifacient might also interfere with the implantation of her/his circulating tumor cells (CTCs) into endothelium, thus the abortifacients may be a good class of cancer metastasis chemopreventives for preventing cancer metastatic cascade from the launch. To test the hypothesis, we started with the most potent abortifacient mifepristone (RU486) that is approved for marketing in more than 55 countries and used by hundreds of millions of women world-wide [4]. Mifepristone is recently used by both genders for long-term psychotic depression or cancer chemotherapy [5–7]. Although its cancer chemotherapeutic trials were not successful as most of the anticancer drugs did, we found and demonstrated that mifepristone and its prime metabolite metapristone were safe and effective in our CTC-based metastatic chemopreventive studies[8–16]. We also found that the extracts from traditional abortion Chinese medicinal plants *Murray paniculata* and *Achyranthes bidentate* produced efficient CTC-based metastatic chemoprevention [17–23]. Although today CTCs are regarded as the root cause of cancer metastases [24], and CTCs separation and identification technologies are very mature [25],drugs for specifically targeting-and-killing CTCs without producing significant side effects are very difficultly to be developed due to the rareness of CTCs in blood. On the other hand, the cancer metastasis chemoprevention strategy that we developed seems feasible to safely and effectively prevent CTC-induced metastases. However, the molecular and cellular mechanisms by which these abortifacients work to prevent CTC-based cancer metastases are basically unknown [26].

The present study was hence designed to interdisciplinarily analyze, in parallel, the biosystem similarities and differences between implantation of blastocyst to uterine endometrium and adhesion-invasion of CTCs to vascular endothelium by using pregnancy-associated hormones 17β-estradiol (E2) and progesterone (P4), and abortifacients mifepristone and metapristone as pharmacological analysis tools. Based on embryo development, we screened key biomarkers that were highly related to embryo development, such as sLex, integrins, E-cadherin, ICAM-1, and CD44. Through systematic analysis, we compared the static and dynamic changes of the two systems in the expression of these key biomarkers, which provides a theoretical basis for contraceptives to prevent tumor metastasis. We hope that the data or the novel idea presented here could open a new horizon in cancer research and treatments, namely, cancer metastasis chemoprevention, to prevent the seeds (CTCs) from gemmating on soil (distant endothelium).

## Materials and methods

### Cell lines and culture

The human uterine endometrial RL95-2 cell line (CRL-1671) was purchased from ATCC, and cultured in RPMI-1640 (Hyclone Inc.) medium containing 10% fetal calf serum (FBS; Gibco), 100 units/mL penicillin and 100 µg/mL streptomycin (Genview Inc.). The human chorionic cell line JEG-3 (HTB-36, ATCC) was provided from Fujian Maternity and Child Care Center, China. The breast cancer cell line MCF-7 (HTB-22, ATCC) was obtained from ATCC and used as a model of circulating tumor cells (CTCs). Both JEG-3 and MCF-7 cells were cultured in DMEM (Hyclone) medium with 10% FBS and antibiotics (100 units/mL penicillin and 100 µg/mL streptomycin). HUVECs were prepared as described by Lu *et al* [27]. After removal of type I collagenase, cells were maintained in 1% gelatin-coated cell culture flasks in M199 (Gibco Inc.) medium containing 20% FBS, 8 units/mL heparin sodium, 10 ng/mL bFGF (Life Inc.), 100 units/mL penicillin and 100 mg/mL streptomycin. HUVECs were used at no more than six passages. All these cells were cultured in a humidified atmosphere of 5% CO_2_ and 95% air.

### Reagents and antibodies

The primary anti-human or anti-mouse antibodies (CD29-PE, CD49a-PE, CD49b-PE, CD49c-PE, CD49d-FITC, CD49e-PE, CD49f-PE, CD51/61-PE, CD44-APC, CD45-FITC, CD47-PE, CD324-PE, CD325-PE, CD326-PE, CD54-PE, CD106-FITC, CD62E-APC, CD62L-APC, CD15s, isotype controls) and secondary antibodies were all obtained from Becton Dickinson (BD) Pharmingen™. Human interleukin-1 beta (IL-1β) was purchased from Cell Signaling Technology, Inc. 17β-estradiol (E2) and progesterone (P4) were purchased from Shanghai Yuanye biological technology Co., Ltd Mifepristone (RU-486) was purchased from Shanghai New Hualian pharmaceutical Co., Ltd with purity > 98%. Metapristone was synthesized by our laboratory with purity > 98%. Calcein-AM was obtained from Dojindo Molecular Technologies, Inc.

### Growth inhibition assay

The growth inhibition of different cells was investigated by the MTT assay as we previously described. Cells were seeded on 96-well plates (1 ×10^4^ cells per well). After 24 h incubation, the culture medium was removed and replaced with the medium containing different concentrations of mifepristone/metapristone (1, 10, 20, 50, and 100 µM). After an additional 24 h of incubation at 37^o^C, the cells were washed with PBS and incubated with 10 µL of 3-(4, 5-dimethylthiazol-2-yl)-2, 5-diphenyltetrazolium bromide (MTT; Genview) (5 mg/mL) and 90 µL of the medium without phenol red for another 4 h. Then 100 µL of dimethyl sulfoxide (DMSO) was added to each well to dissolve the formazan crystals. Cell viability was determined by detecting the absorbance at 570 nm in a microplate reader (Tecan Infinite 200 PRO, Switzerland). All experiments were completed in quadruplicate.

### FACS analysis

Expression of cell-surface ICAM-1, VCAM-1, E-selectin, L-selectin, E-cadherin, N-cadherin, CD45, CD47, sLex, EpCAM, integrin α1, α2, α3, α4, α5, α6, αvβ3 and β1 was measured by flow cytometry. Briefly, cells were plated in 6-well plates, grew to 80% confluence, and then incubated with different concentrations of mifepristone or metapristone. After incubation for 24 h, cells were dissociated with EDTA-free trypsin (Genview) from 6-well plates and 5 × 10^5^ cells were incubated with antibodies for 20 min at 4^o^C in dark. After 3 times of washing with PBS, expression of cell surface biomarkers was analyzed by a BD FACSDiva software. Isotype control IgG was set to replace the primary antibody as a control for background fluorescence intensity. Positive rate (% expression) was defined as the percentage of cells in the gate to exclude isotype control cells.

### RNA extraction and quantitative real-time PCR

Total RNA was extracted from untreated control cells and mifepristone/metapristone treated cells by using Trizol reagent (Invitrogen) according to the manufacturer’s protocol. mRNA was reversely transcribed by the PrimeScript RT reagent kit (Takara). The levels of mRNA expression were detected by real-time PCR using SYBR Premix Ex Taq (Takara) with the Bio-Rad CFX manager software. The primers were designed by the Oligo Primer Analysis 4.0 software and the sequences were analyzed in BLAST. The sequences were 5′-AGATCTTCTTCTTCAAGGACCGGTT-3′ and 5′- GGCTGGTCAGTGGCTTGG

GGTA-3′ for MMP-2, 5′-CTTTGACAGCGACAAGAAGTGG-3′ and 5′- GGCACTG

AGGAATGATCTAAGC-3′ for MMP-9, 5′-AGACCTACACTGTTGGCTGTGAG-3′ and 5′- GACTGGAAGCCCTTTTCAGAG-3′ for TIMP-1, 5′-ATGCACATCACCCT

CTGTGA-3′ and 5′-CTCTGTGACCCAGTCCATCC-3′ for TIMP-2, 5′-TGCACCAC

CAACTGCTTAGC-3′ and 5′- GGAGGCAGGGATGATGTTCT-3′ for GAPDH, 5′-TGCAGTGTGAGGCTGTGTACA-3′ and 5′- GTGGCCACCTGACGCTCT-3′ for integrinα5, 5′-TTTCGATGCCATCATGCAA-3′, and 5′- ACCAGCAGCCGTGTAAC

ATTC-3′ for integrinβ1. For sample analysis, the threshold was set based on the exponential phase of products, and the software program 2-ΔΔCT was used to analyze the data. The expression level of each gene was normalized by using GAPDH mRNA.

### Adhesion assay

RL95-2 cells were seeded into 96-well plates (5 × 10^5^ cells per well). After forming a monolayer, cells were cultured for 24 h in the presence and absence of mifepristone/ metapristone at different concentrations. Trophoblastic spheroids were prepared by shaking the JEG-3 cells on a gyratory shaker at 90 rpm for 24 h until the diameter of spheroids reached about 100 µm that was similar to the human blastocyst size. Calcein-AM (final concentration 1 µM) was added to the medium in the final half hour. These calcein-AM-labeled JEG-3 spheroids in quadruplicate were delivered to each well with a confluent monolayer of RL95-2 cells, and incubated at 37^o^C in a humidified atmosphere with 5% CO_2_ for 1.5 h. We randomly selected 10 visual fields for each well and took photos under a fluorescence microscope (Zeiss, Germany). The adhesion rate was calculated by the formula: number of adhered JEG-3 spheroids/number of total JEG-3 spheroids delivered [28–30].

HUVECs grown to confluence in 24-well plates were pretreated with IL-1β (1 ng/mL) for 4 h. The calcein-AM-labeled MCF-7 cells were co-cultured with HUVECs monolayers in each well, followed by treatment with mifepristone or metapristone for 1.5 h. The non-adherent MCF-7 cells were removed from the plate by washing with PBS, and the adhered cells were counted under a fluorescence microscope. The formula for calculating the adhesion rate was the same as above.

The experimental method for adhesion of JEG-3 or MCF-7 cells to matrigel was similar to that of the adhesion of MCF-7 cells to HUVECs except that the 24-well plates were coated with 2 µg matrigel and blocked with 2% BSA.

### Western blotting analysis

Cells were grown to 90–95% confluence in 6-well plates, and washed with ice-cold PBS and lysed by 100 µL of RIPA buffer containing PMSF (1mM). Cell extracts were centrifuged at 4^o^C to obtain the supernatants for analysis. Protein concentrations of the samples were measured by the BCA protein assay kit. Equal amounts of protein (20 µg/well) were separated by SDS-PAGE and transferred to the polyvinylidene difluoride membrane. The membrane was blocked with 5% skim milk for 1h at room temperature, and then probed with primary antibodies overnight at 4^o^C. Monoclonal mouse anti-human antibodies against β-actin, MMP-2 and MMP-9 (all 1:1000 dilution) were used followed by an additional 1 h incubation with the appropriate HRP-labeled secondary antibody (1:5000). The target protein expression was detected by an enhanced chemiluminescence kit, and quantified with Bio-Rad Quantity One software analysis system. The total MMP-2 or MMP-9 expression was normalized to β-actin levels.

### Scratch assay

JEG-3 and MCF-7 cells were seeded in 6-well plates and cultured until cell monolayers formed. After scratching with a pipette tip, the cells were incubated with different concentrations of E2 plus P4, mifepristone or metapristone for 24 h. Then cells were photographed by using a fluorescence microscope at 0 h and 24 h after the drug treatment. Quantification of cell migration was performed by measuring the wound closure area using Image J software. Each wound closure area at 24 h was compared with the wound closure area at 0 h, and the wound closure area of the control was set as 1. The experiment was conducted in triplicate.

### Cell morphology assay

Cells were seeded on the 24-well plates. After 80% confluent monolayer was formed, mifepristone or metapristone was added to each well at concentrations of 0 and 100 µM for 24 h, cells were then photographed under a fluorescence microscope.

### Mouse embryonic implantation

Normal Kunming mice purchased from the Shanghai Laboratory Animal Center (Shanghai, China) were housed at 22–25°C and 60% humidity condition with ad libitum access to water and food. Two females and one male were caged together, and the females were checked for the presence of a vaginal plug or spermatozoa in the next morning. If the vaginal plug or spermatozoa was found, the day would be defined as D1, and the females would be orally administered with mifepristone or metapristone once (5 mg/kg), or the vehicle on Day 4, and mice were euthanized on Day 8 of the pregnancy, and the number of embryos implanted on the mouse uterus endometrium was counted after laparotomy. All animal studies were performed in accordance with animal protocol procedures approved by the Institutional Animal Care and Use Committee of Fuzhou University.

### Lung metastatic mouse model

The immunocompetent female C57BL/6 mice were cared as described above. They were orally dosed with 5 mg/kg/day of mifepristone and metapristone, respectively, for 3 days before tail-vein injection of homologous 4T1 mouse breast cancer cells (1×10^5^/mouse). Oral mifepristone or metapristone was continually administered for additional 21 days. The mice were then sacrificed and their lungs were excised. The number of lung surface tumor colonies was counted under a dissecting microscope as we described previously [10, 18].

### Immunohistochemistry

For immunohistochemical analysis, the paraffin embedded tissues were cut into 6 µm sections and stained with MMP-2 antibody as described elsewhere. MMP-2 expression in the lung and embryo tissue was determined using avidin-biotin complex (ABC) method. Stained cells were visualized under light microscope at × 200 magnification.

### Statistical analysis

All experimental data were expressed as the mean ± SEM (n = 3–5). The analysis was carried out by using GraphPad Prism 5. Statistical analysis was performed by using the Student’s t-test and one-way ANOVA.

## Results

### Similarities and differences in surface biomarker expressions among seeding and embedding cells

As a rapid readout of cellular and particle data, flow cytometric analysis provides information on cell size and various cell surface biomarkers by its capacity of simultaneous multicolor analysis. As summarized in Table 1 and Table 2 the embedding cells, i.e., human endometrial cells RL95-2 and human umbilical vein endothelial cells (HUVECs), expressed equivalent high levels of integrin β1, α3, α5, α6, and αvβ3. The differences between the two cell lines were that RL95-2 expressed high levels of ICAM-1, and intermediate levels of E-selectin, whereas, HUVECs expressed intermediate levels of ICAM-1, L-selectin and VCAM-1. On the other hand, both the seeding cells JEG-3 and MCF-7 expressed similar high levels of CD47, EpCAM, integrin β1, α5 and α6, and E-cadherin, as well as similar levels of sialyl Lewis-x (sLex). Both JEG-3 and MCF-7 cells showed very low levels of CD44 and CD45. MCF-7 cells expressed N-cadherin higher than JEG-3 cells did. Therefore, the similarities between the two systems in expressing high levels of integrins, in particular, β1, α5, α6, and αvβ3, EpCAM, sLex, E-cadherin, as well as low levels of CD44 and CD45 constructed the common ground for a drug to work on both systems.

**Table 1:** Similarities and differences in % expressions of surface biomarkers between human endometrial epithelial cells (RL95-2 cells) and human umbilical vein endothelial cells (HUVECs)

Biomarkers	RL95-2	HUVEC
Basic control	1.5 ± 0.4	1.2 ± 0.5
Integrinβ1	97.0 ± 3.3	97.1 ± 0.2
Integrinα1	5.1 ± 3.1	5.8 ± 3.2
Integrinα2	5.2 ± 2.1	4.6 ± 1.4
Integrinα3	90.4 ± 3.5	96.5 ± 2.2
Integrinα5	77.5 ± 13.5	96.8 ± 0.4
Integrinα6	96.0 ± 3.8	92.5 ± 3.6
Integrinαvβ3	82.4 ± 5.6	85.2 ± 11.3
ICAM-1	95.4 ± 4.3	51.4 ± 4.3**
VCAM-1	1.2 ± 0.3	29.3 ± 2.7**
E-selectin	53.2 ± 1.3	5.3 ± 2.7**
L-selectin	2.2 ± 0.3	46.3 ± 3.2**

**, *P* < 0.01, compared with the data of RL95-2

**Table 2:** Similarities and differences in % expressions of surface biomarkers between human embryonic cells (JEG-3 cells) and human breast cells (MCF-7 cells).

Biomarkers	JEG-3	MCF-7
Basic control	1.2 ± 0.3	1.5 ± 0.7
sLex	50.5 ± 13.5	59.7 ± 11.2
CD47	80.4 ± 14.2	93.2 ± 2.1
EpCAM	87.4 ± 7.9	86.7 ± 9.5
Integrinα5	75.2 ± 5.9	83.7 ± 5.4
Integrinα6	84.3 ± 7.2	90.3 ± 5.7
Integrinβ1	86.0 ± 8.4	92.1 ± 6.7
Integrinα4	5.3 ± 1.5	73.7 ± 3.3**
CD44	7.6 ± 2.3	4.2 ± 2.0
CD45	4.2 ± 1.5	6.7 ± 2.1
E-cadherin	83.2 ± 11.2	85.6 ± 8.5
N-cadherin	13.3 ± 5.1	64.4 ± 5.2**

**, *P* < 0.01, compared with the data of JEG-3

### Similarities and differences in cell morphology and viability affected by abortifacients

**Fig. 1:**
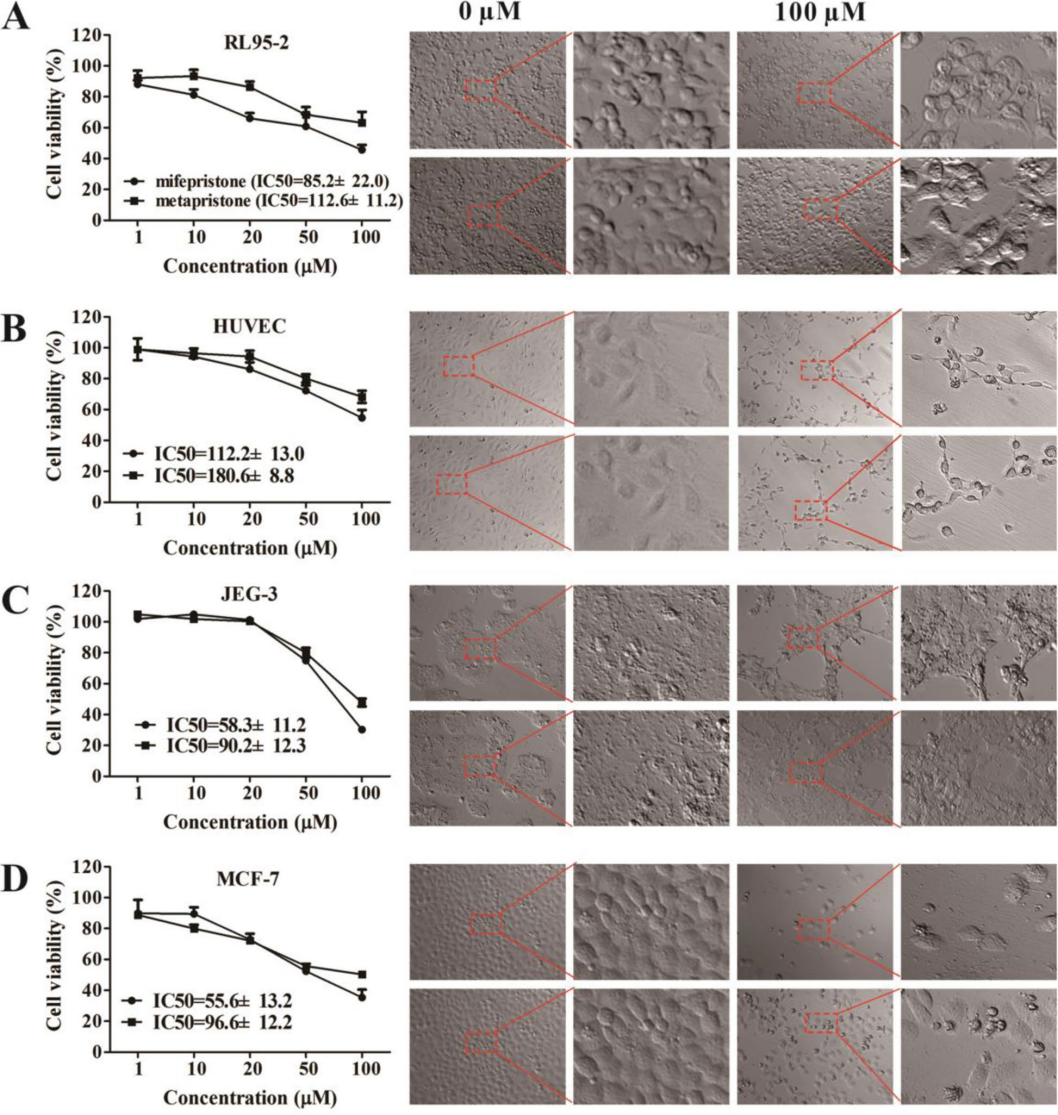
Effects of mifepristone and metapristone on viability and morphology of the tested cell lines. Effects of mifepristone and metapristone on cell viability (left) and morphology (right; upper two panels: mifepristone; lower two panels: metapristone) of endometrial RL95-2 (**A**), endothelial HUVEC (**B**), embryo JEG-3 (**C**), and breast MCF-7 (**D**). The red insets are the amplified (200 folds) photos of the lefts. Some changes in cell morphology were found at 100 µM of drugs. Note, in general, the IC50s of metapristone are higher than those of mifepristone. Data are the mean ± SEM (*n* = 4).

As shown in Fig. 1, in general, both mifepristone and metapristone had cytostatic effects, but mifepristone had IC_50_ values (ranging from 50–112 µM) lower than metapristone (ranging from 90–180 µM) on the four cell lines after 24-h treatment, which indicating that the mifepristone is more cytotoxic. When concentrations of mifepristone and metapristone reached 100 µM, the cell bodies of RL95-2, HUVECs, JEG-3, and MCF-7 became smaller. These seeding trophoblast cells JEG-3 and cancer cells MCF-7 were more sensitive to mifepristone and metapristone than the embedding endometrial cells RL95-2 and HUVECs. There were differences in microscopic morphology between RL95-2 and HUVECs: HUVECs exhibited typical cobblestone growth pattern and became tightly packed but showed no tendency to overlap or overgrow, whereas, endometrial RL95-2 consisted of a single layer of columnar epithelium, and showed orientation outward for interaction with blastocysts.

### Effects of sex hormones and abortifacients on migration of blastocyst JEG-3 and cancer MCF-7 cell lines

**Fig. 2:**
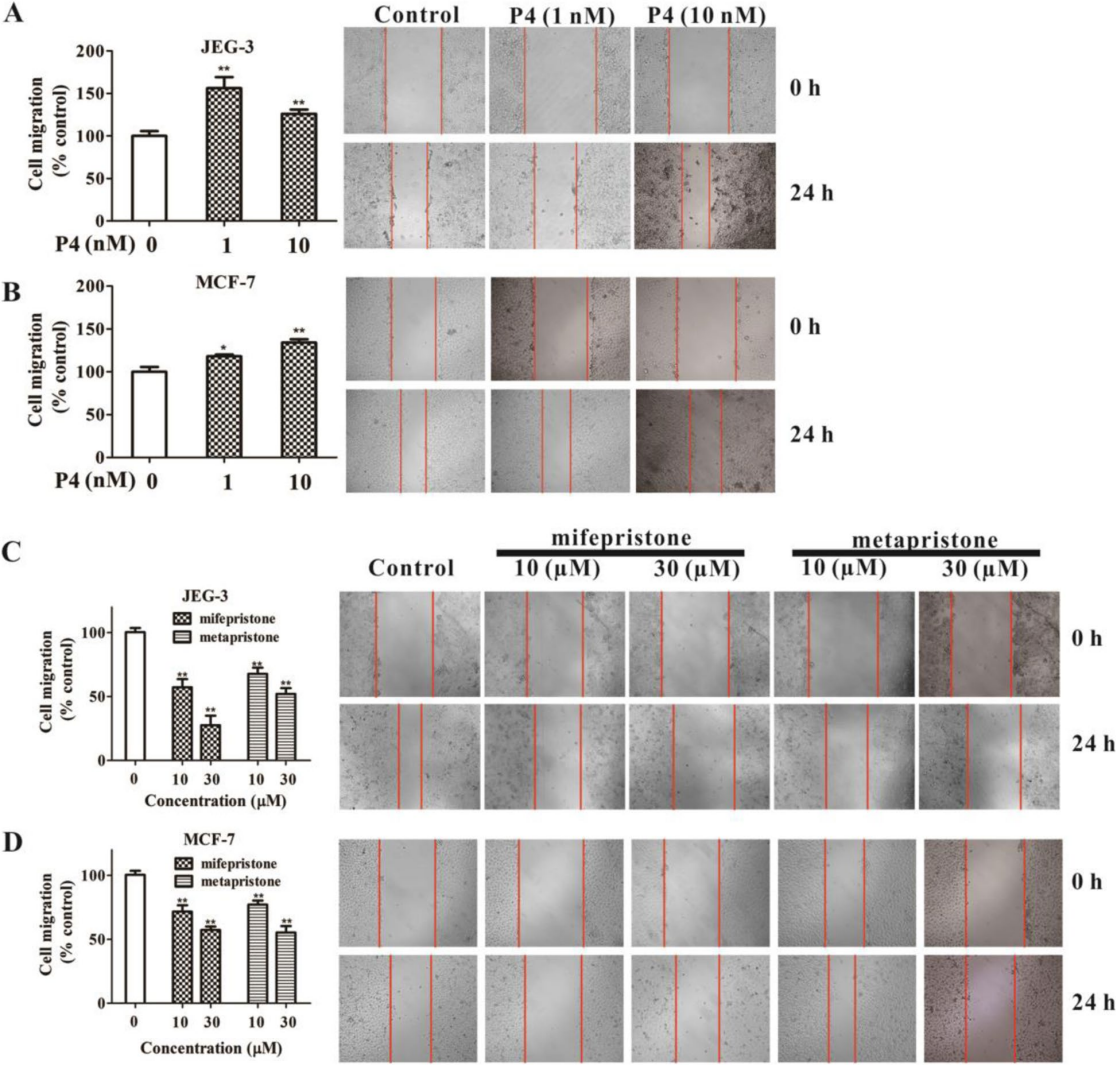
Effects of E2 (10 nM) plus P4, mifepristone and metapristone on the cell mobility of embryo JEG-3 and breast MCF-7 cell lines. Microscopic (right) and quantitative analyses (left) of effects of E2 (10 nM) plus P4 (1, 10 nM) on mobility of JEG-3 (**A**) and MCF-7 (**B**) cells at 0 and 24 h after scratching the monolayer. Microscopic (right) and quantitative analyses (left) of effects of low concentrations of mifepristone and metapristone on mobility of JEG-3 (**C**) and MCF-7 (**D**) cells at 0 and 24 h after scratching the monolayer. Each bar represents the mean ± SEM (*n* = 3); *, *P* < 0.05; **, *P* < 0.01, compared with the control

After the seeding JEG-3 and MCF-7 cells reached confluent, we applied the scratch assay to these seeding cells in the presence and absence of 17ß-estradiol (E2) plus progesterone (P4), mifepristone and metapristone at varying concentrations, and observed the effects of these drugs on spatiotemporal migration of these cells. After 24-h treatment, the constant low concentration of E2 (10 nM) plus P4 promoted migration of JEG-3 and MCF-7 cells. JEG-3 cells seemed to be more sensitive than MCF-7 cells to the low concentrations of hormones (Fig. 2A and B). By contrast, abortifacients mifepristone and metapristone showed concentration-dependent inhibition on migration of this two cell lines. Since the inhibitory concentrations of the abortifacients (10, 30 µM) were lower than their IC_50_, the inhibition should not be derived from cell killing effect of the drugs (Fig. 2C and D).

### Mifepristone and metapristone suppress hetero-cellular adhesion

**Fig. 3:**
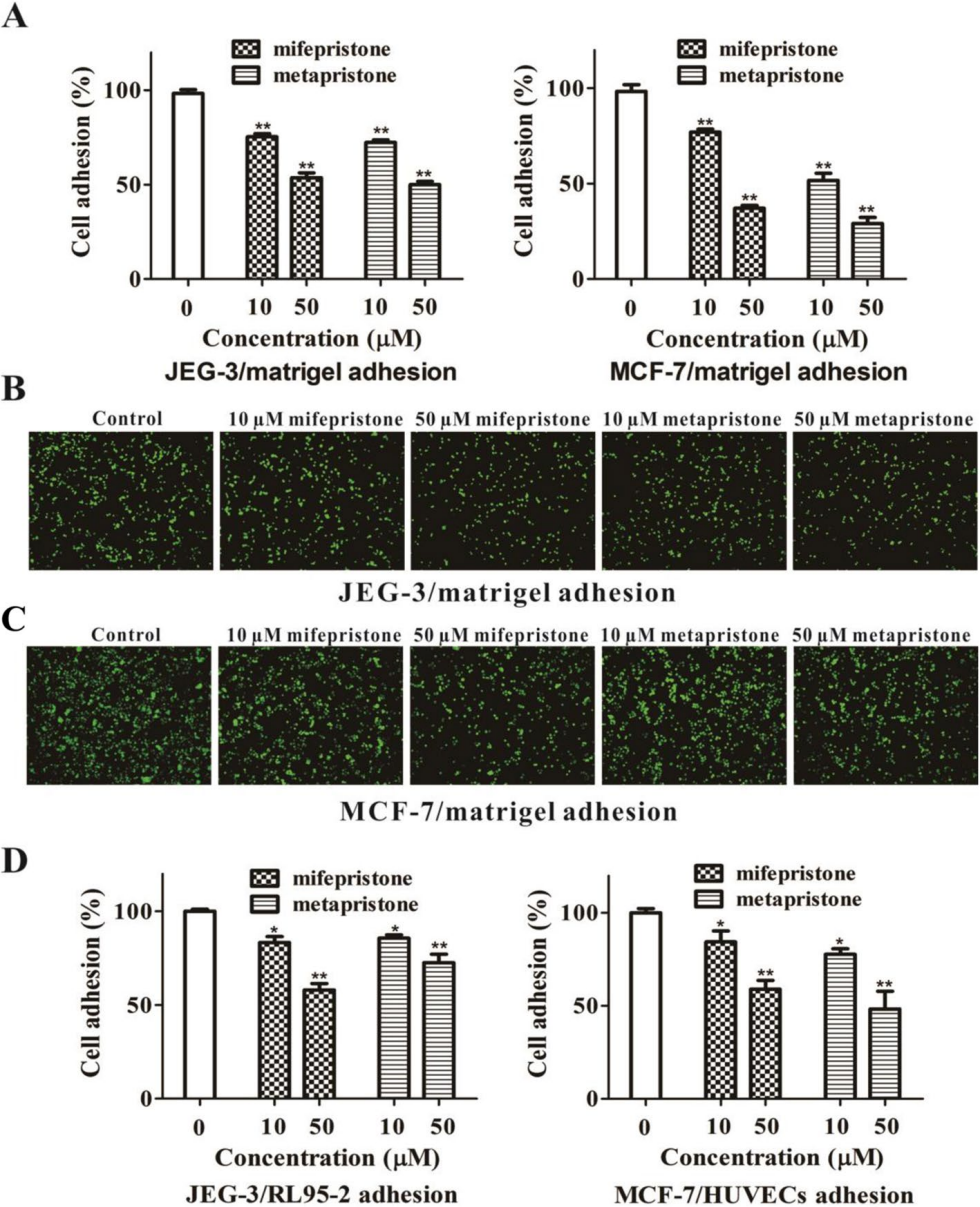
Effects of mifepristone and metapristone on adhesion of embryo JEG-3 and breast MCF-7 cells to matrigel, embryo JEG-3 spheroids to endometrial RL95-2 cells, or breast MCF-7 cells to endothelial HUVECs. Quantitative analysis of the inhibition by mifepristone and metapristone on adhesion of JEG-3 cells (**A**) and MCF-7 cells (**B**) to matrigel. Microscopic illustrations of the inhibition by mifepristone and metapristone on adhesion of JEG-3 cells (**C**) and MCF-7 cells (**D**) to matrigel; Quantitative analysis of the inhibition by mifepristone and metapristone on adhesion of JEG-3 spheroids to RL95-2 monolayer (**E**), and MCF-7 cells to HUVEC monolayer (**F**). Each bar represents the mean ± SEM (n = 3); *, *P* < 0.05; **, *P* < 0.01, compared with the control

We co-cultured the seeding trophoblastic JEG-3 cells with their embedding endometrial RL95-2 cells, and the seeding breast cancer MCF-7 cells with their endothelial HUVECs to determine effects of the abortifacients on their hetero-cellular adhesion. To quantify the effects, we labeled MCF-7 cells with calcein-AM, and then co-cultured MCF-7 with HUVECs or matrigel for 1.5 h in the presence of different low concentrations of mifepristone or metapristone. After removing the non-adherent cells, the remaining adhered cells were evaluated by counting the fluorescent cells to calculate the adhesion rate. To determine if calcein-AM-labeled trophoblastic cells JEG-3 had adhered to the epithelium, the co-culture system was gently shaken for a few times. If JEG-3 remained adhered to the surface of endometrial RL95-2, they were considered to be successfully attached. The microscopic observation revealed that the human blastocyst JEG-3 (100 µm diameter) attached to the RL95-2 cells with their mural trophoblasts. Mifepristone and metapristone inhibited adhesion of both JEG-3 and MCF-7 cells to matrigel in a concentration-dependent manner (Fig. 3A-D). Mifepristone and metapristone also inhibited the hetero-adhesion between JEG-3 spheroids and RL95-2 cells (Fig. 3E), and between MCF-7 cells and endothelial HUVECs in a concentration-dependent manner (Fig. 3F).

### Effects of sex hormones and abortifacients on expressions of cell adhesion molecules

**Fig. 4:**
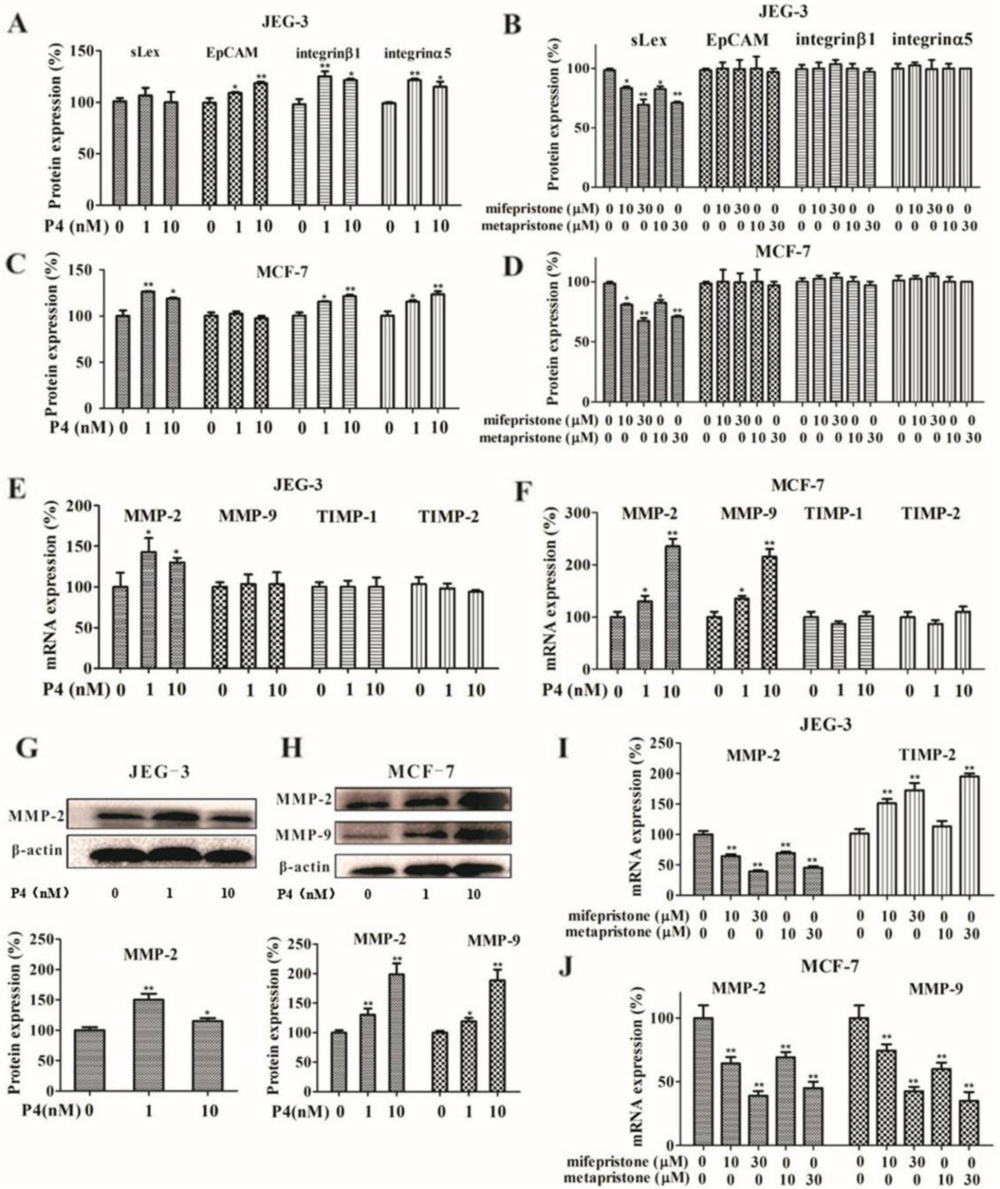
Effects of E2 (10 nM) plus P4, mifepristone and metapristone on the expressions of cell adhesion molecules analyzed by flow cytometry, MMP-2, MMP-9, TIMP-1 and TIMP-2 analyzed by RT-PCR, and western blotting in JEG-3 and MCF-7 cells. E2 (10 nM) plus P4 increased the expression of integrinβ1, integrinα5, and EpCAM in JEG-3 cells (**A**), and increased the expression of integrinβ1, integrinα5, and sLex in MCF-7 cells (**C**). Mifepristone and metapristone decreased the expressions of sLex in both JEG-3 cells (B) and MCF-7 cells (D). Quantitative RT-PCR analysis of expressions of MMP-2, MMP-9, TIMP-1, TIMP-2 by JEG-3 (E) and MCF-7 cells (F) treated with E2 plus P4 at different concentrations. The changes in mRNA levels of each gene induced by E2 plus P4 were shown based on the control. Western blotting and related quantitative analysis showed changes in MMP-2 of JEG-3 cells (G), and MMP-2, MMP-9 in MCF-7 cells (H) induced by E2 plus P4 treatment. RT-PCR analysis showed changes in expressions of MMP-2 and TIMP-2 in JEG-3 cells (I), and of MMP-2 and MMP-9 in MCF-7 cells (J) induced by mifepristone and metapristone. Each bar represents the mean ± SEM (n= 3); *, *P*< 0.05; **, *P*< 0.01, compared with the control

Since integrin α3, α5, α6, β1 and αvβ3, and ICAM-1 were highly expressed on both endometrial cells RL95-2 and HUVECs, we investigated if sex hormones and abortifacients could affect the expression of these molecules on the two kinds of seeding cell lines. Flow cytometric and RT-PCR analysis showed that E2 (at a fixed concentration 10 nM) plus P4, mifepristone and metapristone could not significantly affect the expression of integrin α3, α6 and ICAM-1 on RL95-2 cells and HUVECs. E2 plus P4 increased expression of EpCAM, integrin β1 and α5 in JEG-3 cells (Fig. 4A), and upregulated the expression of sLex, integrin β1 and α5 in MCF-7 cells (Fig. 4C). Mifepristone and metapristone significantly decreased expression of sLex in both JEG-3 and MCF-7 cells without affecting their EpCAM, integrin β1 and α5 levels (Fig. 4B and D).

### Effects of sex hormones and abortifacients on expressions of MMP and TIMP

Matrix metalloproteinases (MMPs) are a family of extracellular matrix degrading proteinases involved in many matrix-degrading abilities in both embryonic implantation and CTC adhesion/invasion. Both JEG-3 and MCF-7 cells were exposed to a fixed concentration of E2 (10 nM) plus P4 (0, 1, 10 nM), and progesterone antagonist mifepristone and metapristone for 24 h. The mRNA and protein expressions of MMP-2, MMP-9, TIMP-1 and TIMP-2 were then examined. In JEG-3 cells, E2 plus P4 increased the mRNA expression of MMP-2 (Fig. 4E). In MCF-7 cells, E2 plus P4 significantly increased the mRNA expression of MMP-2 and MMP-9 (Fig. 4F). The results were further validated by western blotting. The protein expression of MMP-2 in JEG-3 increased after the combination treatment with E2 and P4 (Fig. 4G; upper and lower panels), consistent with the results of RT-PCR. The protein expressions of MMP-2 and MMP-9 in MCF-7 increased significantly with the increased concentrations of P4 (Fig. 4H; upper and lower panels). The treatment of mifepristone and metapristone decreased the mRNA expression of MMP-2, but increased the mRNA expression of TIMP-2 in JEG-3 cells (Fig. 4I). The mRNA expressions of both MMP-2 and MMP-9 decreased significantly in MCF-7 cells in a concentration-dependent manner (Fig. 4J).

### Effects of sex hormones and abortifacients on expressions of integrin β1, α5 and αvβ3

**Fig. 5:**
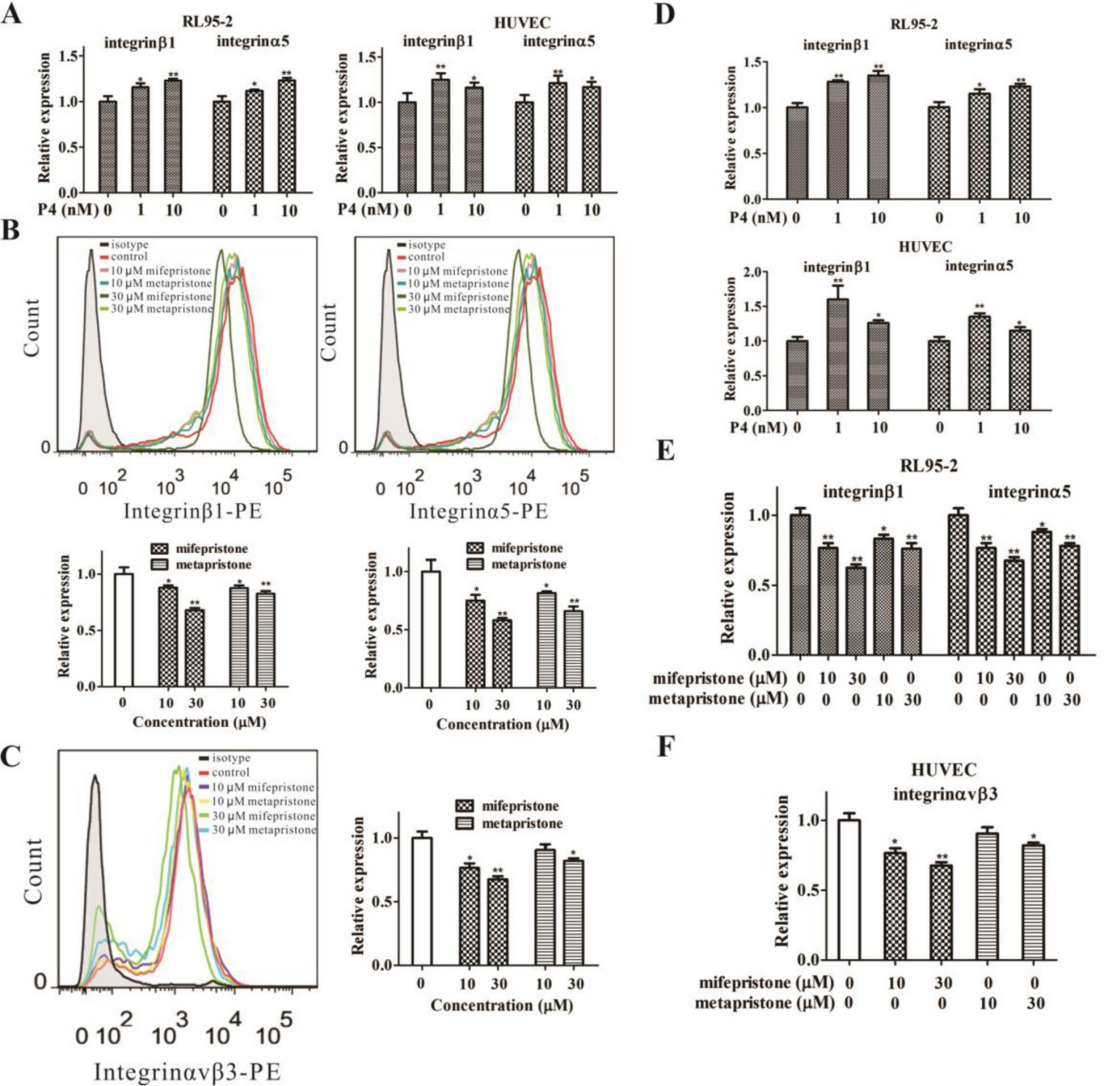
Similarities and differences in morphology and molecular regulations between embryonic implantation to the uterine endometrium and CTC adhesion-invasion to vascular endothelium. **A** Global summary of similarities and differences between blastocyst implantation and cancer cell adhesion and invasion. **B** Morphological differences between blastocyst implantation to the uterine endometrium and CTC adhesion-invasion to vascular endothelium (fluorescence microscope). **C** 17β-estradiol plus progesterone promote, whereas, mifepristone and metapristone inhibit migration and invasion of blastocyst or cancer cells to endometrium or endothelium, respectively, by regulating many cell adhesion molecules involved in the processes

**Fig. 6:**
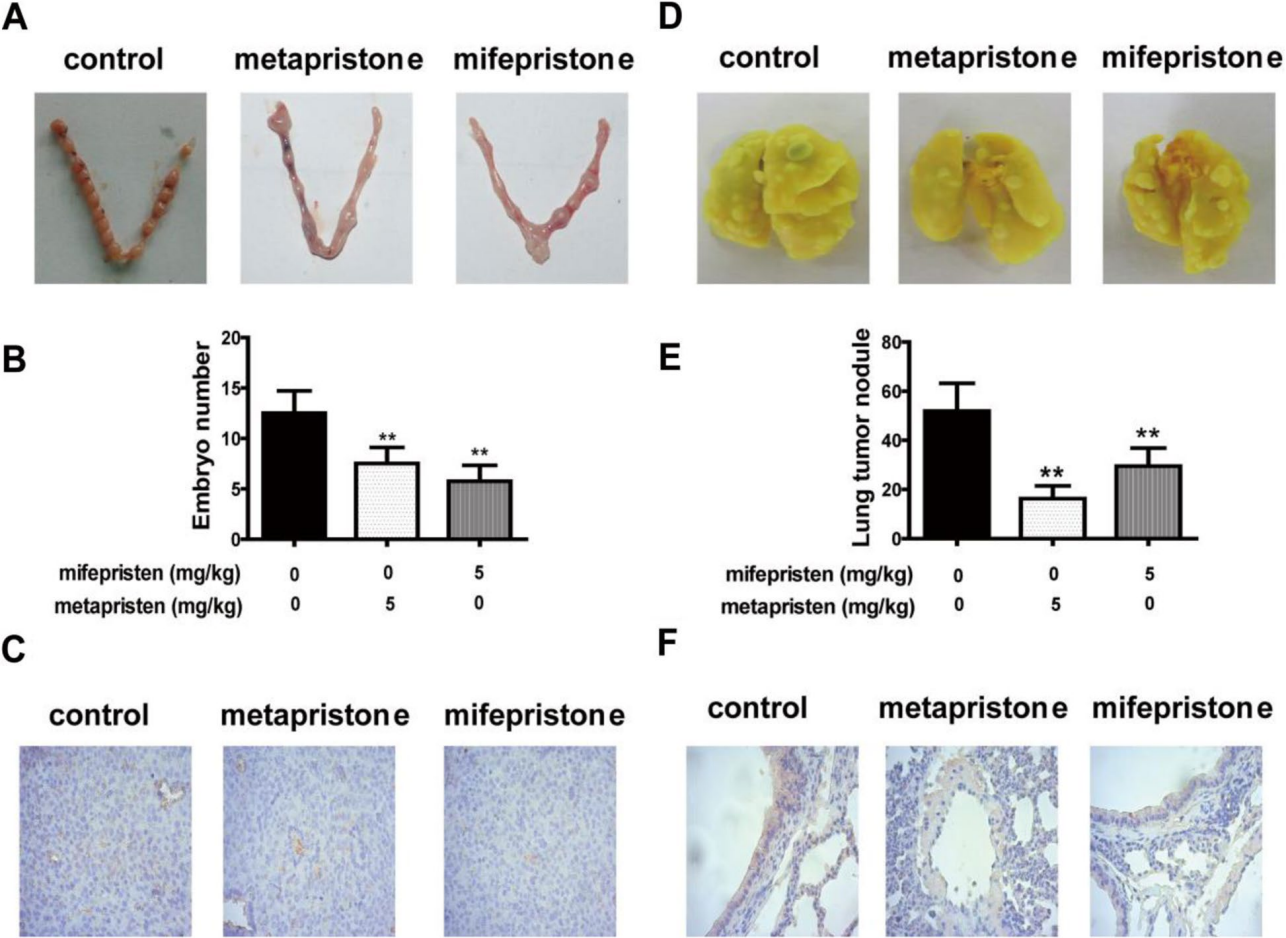
Effects of E2 (10 nM) plus P4, mifepristone and metapristone on the levels of integrinβ1, α5 and αvβ3 expressed by RL95-2 cells and HUVECs, which were analyzed by flow cytometry, RT-PCR. Flow cytometry (**A**, **B**) and RT-PCR (**C**, **D**) analyses showed that E2 plus P4 enhanced the expressions of integrinα5 and β1 in RL95-2 cells and HUVECs, whereas, mifepristone and metapristone inhibited the expressions of integrinα5 and β1 in RL95-2 cells analyzed by flow cytometry (**E**, **F**) and RT-PCR (**G**), integrin αvβ3 in HUVECs analyzed by flow cytometry (**H**) and RT-PCR (**I**). Each bar represents the mean ± SEM (*n* = 3); *, *P* < 0.05; **, *P* < 0.01, compared with the control

Integrins are a family of receptors for various extracellular-matrix ligands that modulate cell–cell adhesion and signal transduction. Each combination of integrin subunits has a unique binding specificity and unique signalling properties. We here investigated effects of sex hormones and abortifacients on expressions of integrin β1, α5 and αvβ3 by the tested cell lines because these integrins usually play important roles in the two implantation systems (Fig. 5). E2 (10 nM) plus P4 (1 or 10 nM) increased protein expressions (Fig. 6A, B) and mRNA expressions (Fig. 6C, D) of integrin α5 and β1 levels in both RL95-2 cells (Fig. 6A, C) and HUVECs (Fig. 6B, D). However, mifepristone and metapristone (10 and 30 µM) significantly decreased protein expressions of integrin β1 (Fig. 6E; upper and lower panels) and integrin α5 (Fig. 6F; upper and lower panels) and the corresponding mRNA levels (Fig. 6G**)** in RL95-2 cells. The same concentrations of mifepristone and metapristone also decreased both protein and mRNA expressions of integrin αvβ3 in HUVECs (Fig. 6H, I).

### In vivo inhibition by mifepristone and metapristone of embryo implantation and CTC adhesion

**Fig. 7:**
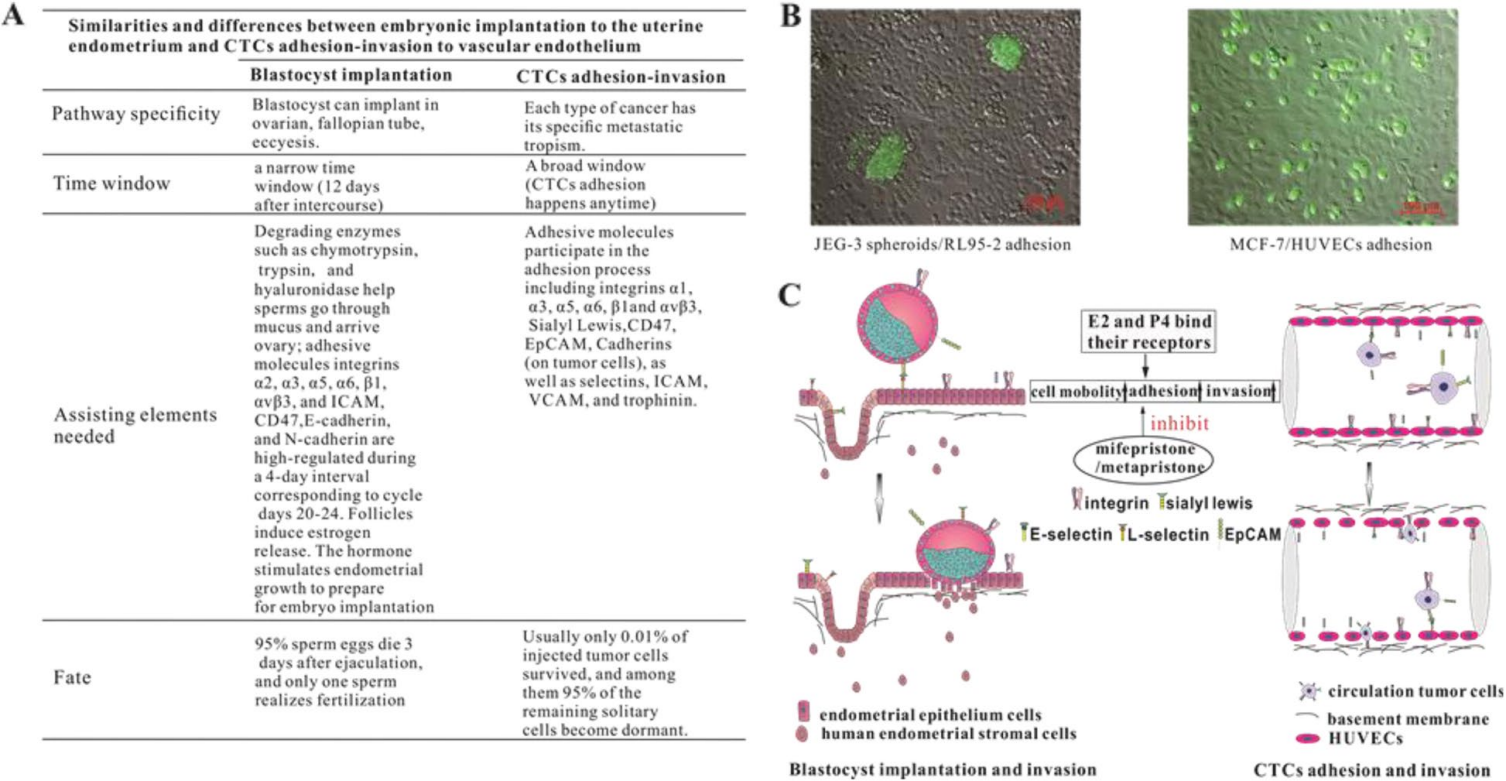
Effects of oral metapristone and mifepristone on embryo implantation to mouse uterine horns of normal female mice and CTC adhesion/invasion to lungs of BALB/C female mice. Photos (**A**) and effects (**B**) of mifepristone and metapristone (both 5 mg/kg given once on Day 4 of pregnancy) on the number of embryos implanted in the mouse uterine horns; (**C**) immunohistochemical staining showed reduction of MMP-2/9 expressed in mouse endometrium after drug treatments. Photos (**D**) and effects (E) of mifepristone and metapristone on lung tumor nodules induced by 4T1 mouse breast cancer cells inoculated (iv) into mouse circulation; (**F**) immunohistochemical staining showed the expressions of MMP-2/9 in mouse embryo and mouse lungs were reduced by metapristone and mifepristone. *n* = 8; *, *P* < 0.05; **, *P* < 0.01, compared with the control

It was the first time that the well-established abortifacients mifepristone and metapristone were tested to explore their effects on both embryonic implantation and CTC implantation, in parallel, to their corresponding embedding tissues. Oral administration of mifepristone or metapristone (5 mg/kg) significantly reduced the number of embryos found in the mouse uterine horn from 12.5 ± 2.1 (control) to 8.5 ± 3.1 (metapristone) and 5.8 ± 1.5 (mifepristone; all *n* = 8 per group; *P* < 0.01) (Fig. 7A-B). On the other hand, the mice treated with mifepristone or metapristone showed a significant decrease in the number of metastatic lung nodules: the mean number of metastatic lung nodules per mouse was 51.9 ± 10.6 (control), 16.3 ± 4.9 (metapristone), 29.5 ± 6.8 (mifepristone; n = 8 per group; *P* < 0.01, Fig. 7D-E). Immunohistochemical staining (Fig. 7C, F) showed that expressions of MMP-2 and MMP-9 in mouse embryos and lungs were reduced by mifepristone and metapristone treatments. It seemed that metapristone was more potent than mifepristone in inhibiting cancer metastasis in the mouse model as we showed before[8–15].

## Discussion

Blastocyst implantation is a complex process including attachment of the blastocyst to the receptive endometrium and invasion of the trophoblastic cells of the conceptus into the endometrium and basement membrane [31]. In the present study, we found that the processes of adhesion, invasion of CTCs to HUVECs and basement membrane were similar to those of the blastocyst implantation. As we analyzed and revealed in the present study, it is the molecular and cellular similarities between the two systems (Figs. 1, 2, 3, 4, and 5) that lead to the in vivo inhibition by mifepristone and metapristone of embryo implantation to uterus and CTC metastasis to lung (Figs. 5 and 7).

Using the in vitro co-culture models of blastocyst/endometrial cells [32] and cancer cells/HUVECs [33], we showed that both mifepristone and metapristone dose-dependently suppressed adhesion of trophoblastic cells JEG-3 to endometrial cells RL95-2 and cancer cells MCF-7 to HUVECs, as well as adhesion of JEG-3 and MCF-7 cells to matrigel (Fig. 3). The molecular mechanism by which mifepristone and metapristone inhibited the implantation or adhesion seems to be related to their downregulation of the expression of EpCAM, sLex, CD47 and integrins in the seeding cells JEG-3 and MCF-7 (Fig. 4A-D). These cell adhesion molecules play an important role in both blastocyst implantation and cancer cell adhesion [34–36].

Physiological concentrations of E2 plus P4 significantly increased expression of integrin α5β1 and MMP2 in JEG-3 cells. The increased expression of MMP-2 was in line with integrin α5β1 at the same time (Fig. 4E, G, I). Matrix metalloproteinase MMP-2, MMP-9, integrinα5β1 and α1β1 played important roles in trophoblast invasion [37]. In the present study, we found that E2 plus P4 increased the expression of MMP and integrin α5β1, and the interactions between MMPs and integrin α5β1 may keep MMP-2 in a proteolytically active form on the cell surface during the migration process of JEG-3 cells. In MCF-7 cells, E2 plus P4 promoted the expression of both MMP and integrin α5β1, thereby increasing the invasive potential of MCF-7 cells (Fig. 4F-H) [38]. E2 plus P4 can also increase activity of integrin αvβ3 which serves as a receptor for MMP-2 to facilitate MMP-2 expression in a functionally active form to promote cell migration. Integrin αvβ3 could simultaneously bind both MMP-2 and collagen fragments [39].

The combined participation of integrins and MMPs in cell migration and invasion is required for invasion of tumor cells into surrounding connective tissues followed by intravasation and extravasation from blood vessels, and metastasis to distant organs [40]. The MMP-2 cleaves ECM proteins fibronectin (FN) and vitronectin (VN) into small fragments to increase the binding of both JEG-3 and MCF-7 cells to FN and VN fragments by their receptors, integrin α5β1 and αVβ3 [41]. By contrast, abortifacients mifepristone and metapristone restrain the invasion, migration and adhesion of both the seeding cells JEG-3 and MCF-7.

As for the embedding cells of endometrial RL95-2 and HUVECs, we showed high levels of β1, α3, α5 and α6 integrins (Fig. 6). ICAM-1 expresses on the two cell lines. E2 (10 nM) plus P4 at different concentrations significantly increased mRNA and protein expression of integrin α5β1 in both RL95-2 and HUVEC cell lines (Fig. 6C-F). Various integrins (α5β1, αvβ3) and FN are expressed by the endometrium [42, 43]. FN is known to induce MMP-2 expression through an integrin-mediated signal transduction pathway in endometrium [44], and the combination of E2 and P4 increased MMP-2 and MMP-9 activity [45]. As for endothelial cells, both fibronectin and vitronectin induce MMP-9 expression via the AP-1-activating signaling pathways by combining with integrin α5β1/αvβ3 in endothelial cells [46]. Our data and others suggest that E2 plus P4 promote the expression of both integrin α5β1 and MMP in endometrial RL95-2 and vascular endothelial HUVECs, and these MMPs promote the invasion and adhesion of both JEG-3 and MCF-7 cells, whereas, mifepristone and metapristone inhibit the expression. Using normal female mice and BALB/C female mice, for the first time, we demonstrated in vivo that abortifacient mifepristone and metapristone could inhibit in parallel both embryo implantation to mouse uterine horns and CTC adhesion/invasion to mouse lungs (Fig. 7).

## Conclusions

In summary, it is the first time that we revealed the similarities and differences between the embryonic implantation system and CTC adhesion/invasion system by using co-culture systems of trophoblastic cells JEG-3/endometrial cells RL95-2 and breast cancer cells MCF-7/endothelial HUVECs, as well as various molecules that play important roles in regulating the implantation and adhesion/invasion microenvironments. (Fig. 6). The seeding cells JEG-3 and MCF-7 express high levels of sLex, CD47, EpCAM, integrins α5, α6, β1, and E-cadherin. The embedding cells RL95-2 and HUVECs exhibit high levels of integrin α3, α5, α6, β1, αvβ3, and ICAM-1. Low concentrations of E2 plus P4 promote migration and invasion of JEG-3 and MCF-7 cells via upregulating EpCAM, integrins, MMPs in JEG-3 and MCF-7. By contrast, low concentrations of abortifacients mifepristone and metapristone significantly inhibit migration and invasion of seeding cells JEG-3 and MCF-7 to their embedding cells RL95-2, HUVECs and matrigel, via downregulating sLex, MMPs in JEG-3 and MCF-7, integrins in RL95-2 and HUVECs. The interactions between seeding CTCs or embryos and their microenvironments “niche” at a correct spatiotemporal point are important for the seeds to gemmate into the soil [47]. The similarities between the two systems provide fundamentals for abortifacients to intervene CTC adhesion/invasion to the distant metastatic organs in vivo. The present study offers the rationale to explore the huge abortifacient treasure to identify the safe and effective cancer metastatic chemopreventive agents to inhibit the CTC-based cancer metastasis. The new strategy may revolutionize the future cancer research and treatment.

## Data Availability

The datasets used and/or analysed during the current study are available from the corresponding author on reasonable request.

## Abbreviations

CTCs: Circulating tumor cells
JEG-3: The human chorionic cell line JEG-3
MCF-7: The breast cancer cell line MCF-7
RL95-2: H uman uterine endometrial RL95-2 cell line
HUVECs: Human umbilical vein endothelial cells
FBS: Fetal calf serum
MTT: Methyl thiazolyl tetrazolium
DMSO: Dimethylsulfoxide
PCR: Polymerase chain reaction
ABC: Avidin-biotin complex
MMPs: Matrix metalloproteinases
TIMP: Tissue inhibitor of metalloproteinases
VN: Vitronectin
FN: Fibronectin
ECM: Extracellular matrix
PBS: Phosphate buffered saline
