# Correction to: Embedding similarities between embryos and circulating tumor cells: fundamentals of abortifacients used for cancer metastasis chemoprevention

**DOI:** 10.1186/s13046-021-02132-0

**Published:** 2021-10-22

**Authors:** Jie Wang, Xiaobo Yu, Huayi Peng, Yusheng Lu, Shuhui Li, Qing Shi, Jian Liu, Haiyan Dong, Vladimir Katanaev, Lee Jia

**Affiliations:** 1 College of Materials and Chemical Engineering, Minjiang University, Fuzhou, 350108 China; 2 Cancer Metastasis Alert and Prevention Center, College of Chemistry, Fujian Provincial Key Laboratory of Cancer Metastasis Chemoprevention and Chemotherapy, Fuzhou University, 350108 Fuzhou, P.R. China; 3 Fujian Provincial Key Laboratory of Inspection and Quarantine Technology Research/ Technology Center of Fuzhou Customs, Fuzhou, 350108 China; 4 Fujian Key Laboratory for Translational Research in Cancer and Neurodegenerative Diseases Institute for Translational Medicine, School of Basic Medical Sciences, Fujian Medical University, Fuzhou, 350108 China; 5 Translational Research Center in Oncohaematology, Department of Cell Physiology and Metabolism, Faculty of Medicine, University of Geneva, Geneva, Switzerland

**Correction to: J Exp Clin Cancer Res 40, 300 (2021)**

**https://doi.org/10.1186/s13046-021-02104-4**

Following publication of the original article [1], an error was found in the Author’s contributions section. The correct details should read as:

“Lee Jia designed, wrote and supervised the study. Xiaobo Yu, Jie Wang, Huayi Peng, Qing Shi performed all experiments. Yusheng Lu, Shuhui Li, Jian Liu, Haiyan Dong, and Vladimir Katanaev participated in data analyses, or technology consultations.”

Furthermore, author affiliations and their corresponding affiliation details are updated to:

Jie Wang^1†^, Xiaobo Yu^2†^, Huayi Peng^3†^, Yusheng Lu^1^, Shuhui Li^2^, Qing Shi^2^, Jian Liu^2^, Haiyan Dong^4^, Vladimir Katanaev^1,5^ and Lee Jia^1,*^

^1^College of Materials and Chemical Engineering, Minjiang University, 350108 Fuzhou, China.

^2^Cancer Metastasis Alert and Prevention Center, College of Chemistry; Fujian Provincial Key Laboratory of Cancer Metastasis Chemoprevention and Chemotherapy, Fuzhou University, 350108 Fuzhou, P.R. China.

^3^Fujian Provincial Key Laboratory of Inspection and Quarantine Technology Research/ Technology Center of Fuzhou Customs, 350108 Fuzhou, China.

^4^ Fujian Key Laboratory for Translational Research in Cancer and Neurodegenerative Diseases Institute for Translational Medicine, School of Basic Medical Sciences, Fujian Medical University, 350108 Fuzhou, China.

^5^Translational Research Center in Oncohaematology, Department of Cell Physiology and Metabolism, Faculty of Medicine, University of Geneva, Geneva, Switzerland.

The correction does not have any effect on the results or conclusions of the paper. The original article has been corrected.
